# Endothelial PERK-ATF4-JAG1 axis activated by T-ALL remodels bone marrow vascular niche

**DOI:** 10.7150/thno.67710

**Published:** 2022-03-21

**Authors:** Cui Liu, Qiuyun Chen, Yinghui Shang, Lechuang Chen, Jay Myers, Amad Awadallah, Jinger Sun, Shuiliang Yu, Katharine Umphred-Wilson, Danian Che, Yingtong Dou, Luoyi Li, Pamela Wearsch, Diana Ramírez-Bergeron, Rose Beck, Wei Xin, Ge Jin, Stanley Adoro, Lan Zhou

**Affiliations:** 1 Department of Pathology, Case Western Reserve University, Cleveland, OH 44106, USA; 2 Department of Blood Transfusion, the Third Xiangya Hospital, Central South University, Changsha, 410013, China; 3 Department of Biological Sciences, School of Dental Medicine, Case Western Reserve University, Cleveland, OH 44106, USA; 4 Department of Pediatrics, Case Western Reserve University, Cleveland, OH 44106, USA; 5 Department of Pathology, University Hospitals Cleveland Medical Center, Cleveland, OH 44106, USA; 6 Department of Medicine, Case Western Reserve University, Cleveland, OH 44106, USA

**Keywords:** T-ALL, Vascular niche, UPR, PERK, JAG1

## Abstract

The endoplasmic reticulum unfolded protein response (UPR) is a conserved adaptive signaling in ER homeostasis and has emerged as critical in highly proliferating cells and potential treatment target for acute T-cell lymphoblastic leukemia (T-ALL).

**Methods:** in this study, we assessed the transcriptomic and phenotypic alterations in UPR response of the bone marrow endothelial cells (ECs) in mice engrafted with T-ALL and in bone marrow specimens from patients who have T-ALL. We used PERK inhibitor and generated endothelial specific PERK knockout mice to study the impact of PERK on leukemia progression and hematopoiesis. We performed chromatin immunoprecipitation (ChIP) to study the mechanistic regulation of JAG1 by ATF4. We characterized small extracellular vesicles (SEV) from leukemia-developing mice and studied the effect of SEVs on EC function.

**Results:** we found that T-ALL development induced a robust activation of protein kinase RNA-like endoplasmic reticulum kinase (PERK)-dominant UPR in the bone marrow endothelial vascular niche. The activation of PERK-eIF2a-ATF4 axis remodels the vascular niche, upregulates angiogenic factors including VEGFα and ATF4-regulated JAG1, and suppresses the expression of SCF and CXCL12, which are important to HSC maintenance and regeneration. Further, targeting endothelial PERK significantly improved T-ALL outcome. EC-specific deletion of PERK abolished the aberrant JAG1 up-regulation, improved HSC maintenance, promoted leukemia apoptosis, and improved overall survival. Finally, we showed that small extracellular vesicles are critical mediators of endothelial PERK-eIF2a-ATF4 activation and JAG1 up-regulation in leukemia. Corroborating animal model studies, activation of PERK-ATF4-JAG1 is prominent in human T-ALL bone marrow and T-ALL xenografts.

**Conclusion:** our studies thus revealed for the first time that the leukemia-initiated PERK-ATF4-JAG1 axis plays a critical role in the remodeling of the bone marrow vascular niche and that targeting vascular niche UPR is a potential therapeutic opportunity in T-ALL.

## Background

Acute lymphoblastic leukemia (ALL) is the second most common acute leukemia in adults 1, 2. T-ALL patients have poorer prognoses and higher relative risk of release or refractory disease than B-ALL patients 3, with at least 20% of pediatric and 40% of adult T-ALL patients eventually relapsing after receiving high-dose multi-agent chemotherapy regimens 4. Re-induction with aggressive chemotherapy or stem cell transplantation is associated with long-term sequelae and overall poor outcomes 5. Although outcomes for relapsed/refractory B-cell acute lymphoblastic leukemia (B-ALL) have been improved by monoclonal antibody therapy and chimeric antigen receptor (CAR) T-cell immunotherapy, this option has been challenging to apply in T-ALL 6. Therefore, there is a need to understand T-ALL propagation mechanisms and to improve treatment options.

Identifying the relationship between leukemia and the bone marrow (BM) microenvironment can refine targeted approaches to T-ALL. Diffuse infiltration of the BM and CNS expansion through marrow factors is an essential step for T-ALL propagation 7, 8. Hematopoietic stem cells (HSCs) reside in the specialized compartment called the stem cell niche. Reconstruction of the HSC niche into a leukemia sanctuary site is recognized as a mechanism for leukemia-initiating cells to survive, enabling relapse in acute myeloid leukemia 9, 10. Similarly, in animal models of human T-ALL, lymphoblast leukemia cells suppress normal hematopoiesis through two critical niche components: osteoblasts and the peri-vascular region 11. Aberrant activation of Notch in the stroma suppresses normal hematopoietic stem cell and progenitor cell (HSPC) proliferation. It suppresses osteoblast differentiation associated with decreased CXCL12 elaboration. However, the underlying mechanism by which niche construction affects vascular niche endothelial cells (ECs) and suppresses normal residual hematopoiesis remains unknown.

In this study, we identified that endothelial unfolded protein response (UPR) regulates leukemia-induced vascular niche remodeling and contributes to leukemia progression and suppression of hematopoiesis through the involvement of endothelial protein kinase RNA-like endoplasmic reticulum kinase (PERK), eukaryotic initiation factor 2 (eIF2), activating transcription factor 4 (ATF4), and Jagged canonical Notch ligand 1 (JAG1). Using a mouse model of T-ALL, we found that the PERK-eIF2-ATF4-JAG1 axis is robustly activated in the BM of mice developing leukemia, a process dependent on the activity associated with leukemia released small extracellular vesicles (SEVs) that activate PERK. Furthermore, we showed that targeting endothelial PERK improves vascular niche function by inducing leukemia apoptosis and increasing residual hematopoietic progenitors.

## Materials and Methods

### Mice

The animal research described in this article was approved by the Institutional Animal Care and Use Committee. Floxed PERK mice (PERK^F/F^) and VE-cadherin^ERT2-Cre^ mice (obtained from R. Adams) 12, 13 were crossed to generate inducible endothelial-specific knockout of PERK mice (PERK^ECKO^ or PERK KO). Deletion of PERK was mediated by 5 consecutive doses of tamoxifen. PERK expression was assessed by anti-PERK (BS-2469R; Bioss Antibodies Inc., Woburn, MA) through FACS analysis. NSG mice (8-12 weeks old) were maintained in the Case Comprehensive Cancer Center Shared Resource Athymic Animal Core.

### Retroviral transplantation and analysis of leukemia mice

Retroviral (pMIG-eGFP-ICN1) transfection was performed as described 11. Secondary leukemia was generated in non-irradiated mice by intravenous injection of primary leukemia splenocytes (0.5-2 X 10^6^). FACS analysis was performed as described 11. Antibodies used for analysis of HSPC (Lin^-^Sca1^+^c-kit^+^) were purchased from BD (San Jose, CA), eBioscience (San Diego, CA), and Biolegend (San Diego, CA) and included CD4 (RM4-5), CD8α (53-6.7), B220 (RA3-6B2), CD11b (M1/70), Gr-1 (RB6-8C5), TER119 (TER-119), c-kit (2B8), and Sca1 (D7). Analysis of HSPC (Lin^-^Sca1^+^c-kit^+^) was achieved by gating on GFP^-^CD45^-^TER119^-^ cells. Antibodies used for analysis of UPR, including CD31 (390), CD45 (30-F11), PERK (C33E10), p-PERK (T980) (16F8), phospho-eIF2α (Ser51) (9721S), ATF4 (D4B8), and JAG1 (D4Y1R) were sourced from eBioscience, Cell Signaling, and Biolegend. For JAG1 staining, BM cells were incubated with JAG1 antibody for 30 min followed by incubation with CD31, CD45, and TER119 antibodies for 20 min. FACS analysis was performed on FACAria I or CytoFLEX flow cytometer after washing cells with PBS. For analysis with p-EPRK, p-eIF2a and ATF, bone marrow cells were incubated with CD31, TER119 and CD45 for 20 min on ice and then fixed and permeabilized for 20 min at room temperature with Fixation/Permeabilization buffer for 20 min at room temperature. Cells were then incubated with the p-PERK, p-eIF2a, or ATF4 antibodies for 15-20 min.

### Cell culture, transfection and chromatin Immunoprecipitation (ChIP)

Mouse immortalized endothelial MS1 cells (MILE SVEN1; ATCC CRL-2279) and BMECs (Cellbiologics, Chicago, IL) were cultured in DMEM (10% FBS) and Complete Endothelial Cell Medium Kit (M1168PF, Cellbiologics). After MS1 or BMECs reached 80% confluent in RPMI-1640 (10% exosome-free FBS, 2mM GlutaMAX^TM^, 1mM sodium pyruvate, 5μM 2-mercaptoethanol and 100 μg/mL penicillin/streptomycin), 1.5 X10^6^ BM cells from WT or leukemic mice were seeded onto the MS1 or the BMECs and co-cultured for 24 h. For PERK inhibition or SEV release blocking studies, cells were treated with 1mM GSK2606414 (EMD Millipore) or 20 µM GK4869 (MilliporeSigma, Burlington, MA) for 24 h, respectively. MS1 cells or BMECs were harvested 24 h later for protein and RNA extraction following negative selection with CD45-Biotin beads. Before siRNA transfection using lipofectamine^TM^ 3000 (Invitrogen, Carlsbad, CA), MS1 cells were co-cultured with ICN1 or control cells (or supernatant) for 24 h. At 72 h post-transfection, cells were harvested and subjected to RNA or protein analysis. To assess if ATF4 binds the CRE region of JAG1 in ECs co-cultured with WT or ICN1 cells, ChIP was performed according to the manufacturer's instruction using the ChIP Assay kit (Millipore, #17-295). After crosslinking, the EC lysates were immunoprecipitated with either rabbit IgG or an anti-ATF4 (D4B8) (Cell Signaling). The JAG1 in complex with ATF4 was detected by the QPCR with the following primers: JAG1F, 5'-GCAGGCCCTCCTCCC-3'; JAG1R, 5'-GTAGGCGCCGCGGTA-3'.

### SEV isolation and characterization

Exosome-free FBS was prepared through centrifugation at 100,000 g overnight. To isolate SEVs from mouse blood, 2 mL of plasma was centrifuged at 400 g for 15 min, diluted with 2 ml PBS followed by centrifugation at 11,000 g for 10 min. After filtration through a 0.22 µm filter, SEVs were pelleted by ultracentrifugation at 100,000 g for 1 h at 4 °C, washed with 10 ml of PBS, and pelleted again at 100,000 g for 1 h. SEVs were quantified using the AChE assay (SBI System Bioscience, Palo Alto, CA) following the manufacturer's instructions. Nanoparticle tracking was used to determine SEV concentration in some experiments using diluted plasma and visualized on the NanoSight NS300. SEVs were also characterized by electron microscopy and western blot 14, 15. Antibodies for blotting include CD9 (ab92726; Abcam, Cambridge, UK), CD63 (ab217345; Abcam), and CD81 (M38; Invitrogen). For *in vitro* tracing, SEVs were labeled using 10 μM CFSE (Thermo Fisher) in PBS for 30 min at 37 °C. 40 x10^8^ / ml CFSE-labeled SEVs were co-cultured with MS1 for 4 h with or without Cytochalasin D (CD). Uptake of CFSE-labeled SEVs was analyzed by FACS.

### Human T-ALL cell studies

Approximately 1 × 10^6^ DND41 cells were IV injected into 6 ~ 8-week-old NOD SCID IL2Rγ null (NSG) mice. Mice were euthanized upon development of signs of leukemia. Peripheral blood and bone marrow were collected and analyzed for the presence of human leukemic cells through human CD45 expression. FACS analysis was performed as described for ICN1 mice.

### Immunohistochemistry

Study of human marrow specimens was approved by the Institutional Research Board (IRB) of the University Hospitals Case Medical Center. The study includes 5non-leukemia control and 4 T-ALL patient BM sections. Expression of p-PERK, p-eIF2a and JAG1 were evaluated by immunohistochemistry (IHC). Sections were incubated with the primary antibodies overnight at 4°C (anti p-PERK, 1:200, Cell signaling; anti-p-eIF2a,1:50, Cell Signaling; anti-JAG1, 1:1000, Sigma, St. Louis, MO). Images were analyzed on an Aperio Image Scope.

### Whole-mount immunostaining

To analyze the BM vascular network, 10 μg each of Alexa Fluor 647 anti-mouse CD31 and Alexa Fluor 647 anti-mouse CD144 (BioLegend) were IV injected 10min before euthanasia as described 16, 17. The sternal bone fragments were fixed in ice cold 4% PFA for 3h. Whole-mount fragments were directly imaged on a Leica SP5 inverted confocal imaging system. Fluorescent light emissions were collected using the internal detectors set to 494 - 538 nm (GFP), 553 - 617 nm (PE), and 644 - 710 nm (AF647). High resolution 3 dimensional (xyz) scans were performed using a Leica 10x objective (N.A. 0.4), with XYZ voxel sizes of 1.5 μm x 1.5 μm x 5 μm. Images were analyzed using Imaris software (Bitplane, Inc, Belfast, UK). Volume data for each image was determined by generating surfaces in Imaris based on the corresponding fluorescent signals. The overall volume of the image was used to normalize the data and to determine percentages.

## Results

### T-ALL Development Induced Up-regulation of JAG1 and Down-regulation of SCF and CXCL12 in ECs

We previously reported that up-regulation of JAG1 in stroma cells is a prominent feature of T-ALL mouse BM Notch activation. In addition, leukemia initiating cells outcompete normal hematopoietic progenitors at the perivascular niche and niche dysfunction leads to gradual depletion of the normal hematopoietic progenitors 11. To study the mechanism by which ALL leukemia induces peri-vascular niche dysfunction, we focused on leukemia-exposed marrow ECs using the established mouse model of T-ALL induced by activated Notch1 (ICN1) 11, 18.

**Figure 1:**
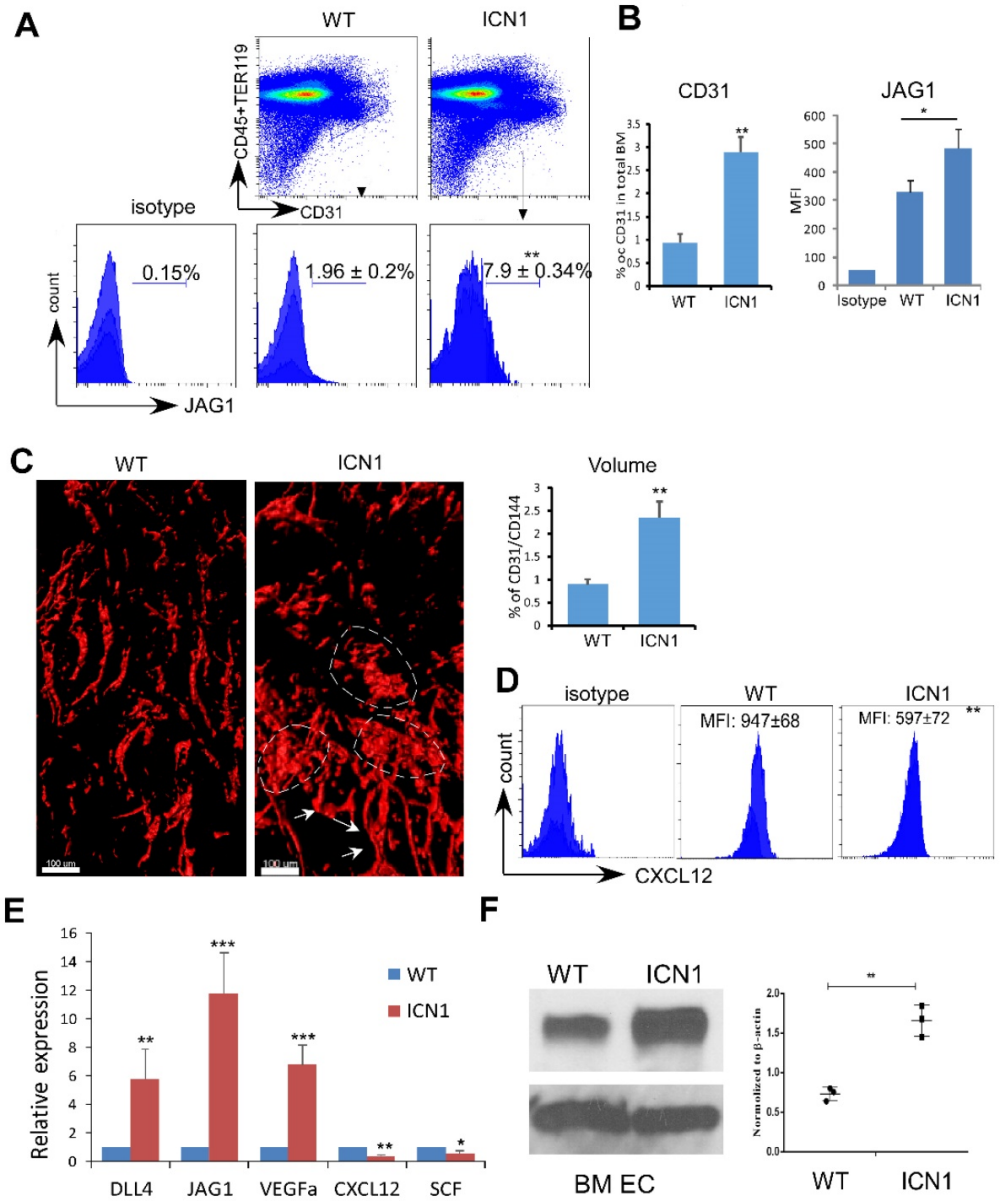
** T-ALL Development Induced Up-regulation of JAG1 and Down-regulation of SCF and CXCL12 in ECs.** (A-B) Representative FACS profile of bone marrow ECs (Lin^-^TER119^-^CD31^+^) and EC-expressing JAG1 (A). Frequency of bone marrow CD31^+^ ECs and mean MFI of JAG1 determined from 4 similar experiments (B). (C) Representative whole-mount imaging of CD31/CD144^+^ bone marrow vasculature in the control WT and ICN1 mice (left panel). Dilated small arteries and coalesced vessels were highlighted by arrows and dotted circles, respectively. Right panels plot the changes in CD31/CD144 volume (%) by whole-mount imaging. (D) Representative EC (CD45^-^TER119^-^CD31^+^) expression of CXCL12 by FACS analysis in control mice or in ICN1 leukemia mice. Mean MFIs were calculated from 4 similar experiments. (E) BMECs were co-cultured with control or ICN1 cells (1.5 X10^6^) for 24h. qRT-PCR of EC expressions of transcripts were standardized for beta-actin and expressed as fold changes relative to those in EC co-cultured with control marrow cells (n=6/group from 2 experiments). (F) Representative western blot (left) with anti-JAG1 of BMECs co-cultured with control or ICN1 cells. Quantification of JAG1 expression normalized to β-actin from 3 similar experiments (right). Data shown in A, B, C (right), D-F are mean ± SD (n=3-6/group from 2-4 experiments). Student t test was performed; *p<0.05; ** p<0.01.

We observed significant induction of JAG1 in the expanded endothelial compartment of the BM of leukemia mice (referred to as ICN1 mice) (Fig. 1A). The BM vascular density assessed by whole-mount CD31 and CD144 labeling or by flow cytometry using CD31 antibody was increased by 162% and 211%, respectively, in ICN1 mice (Fig. 1B & C). In addition, BM vessel architecture was extensively altered through numerous short vessels, dilation of the lumen of small arteries, and amalgamation of vessels (Fig. 1C). Consistent with our previous observation that CXCL12 in the stroma of leukemic mice was suppressed, we found that CXCL12 was also decreased in leukemic BM ECs (Fig. 1D).

**Figure 2:**
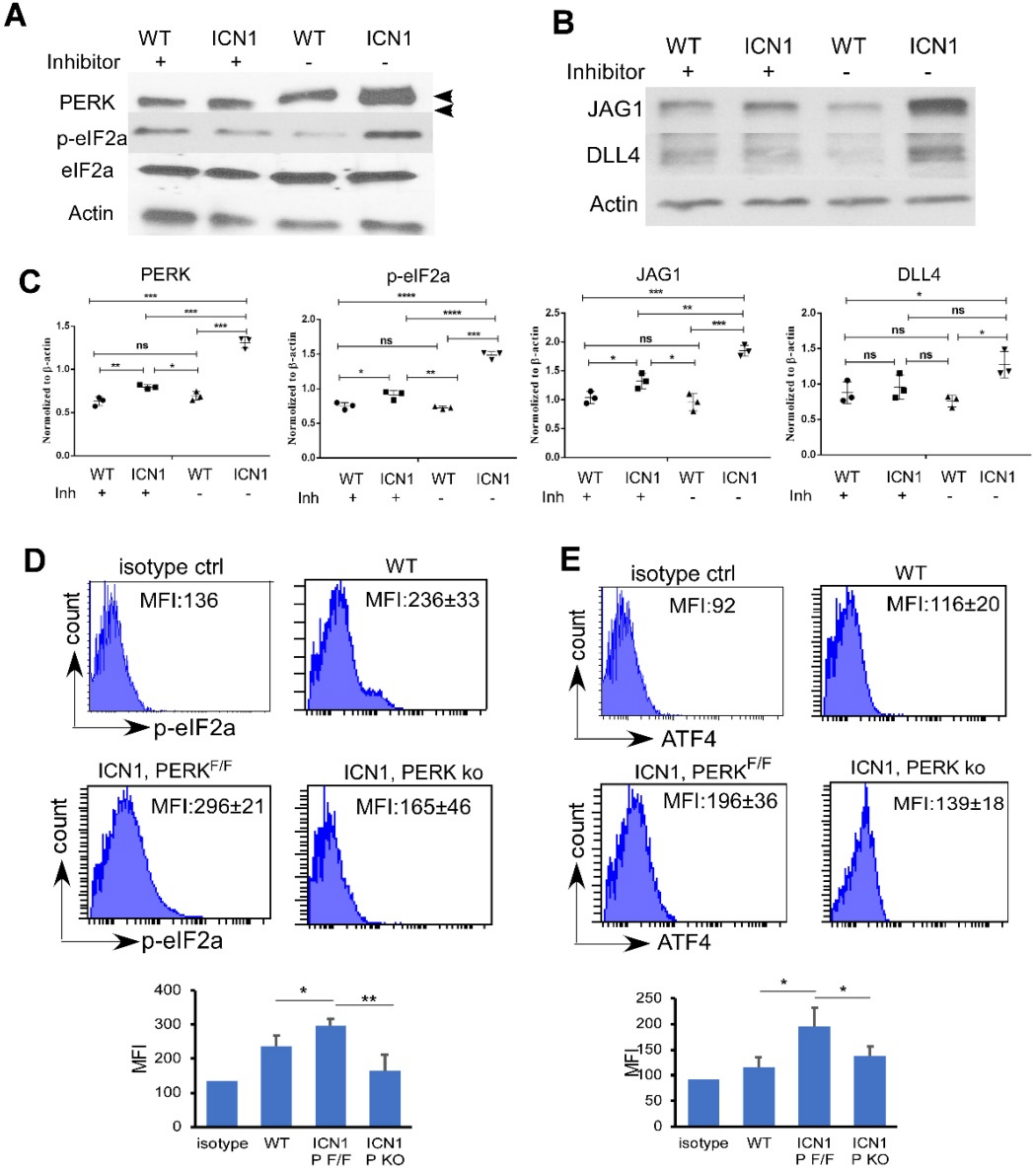
**PERK-eIF2a-ATF4 Activation induced in ECs by ICN1 is Necessary for JAG1 Up-regulation.** (A-B) BMECs were co-cultured with control or ICN1 cells (1.5 X10^6^) for 24h in the presence or absence (GSK control medium) of the PERK inhibitor GSK2606414 (GSK; 1 µM). Representative western blots using antibodies targeting PERK (3192; Cell signaling), p-eIF2α (3398; Cell signaling), total eIF2α (9722; Cell signaling), JAG1 (70109; Cell signaling), DLL4 (21584-1-AP; Proteintech), and β-actin are shown from three similar experiments. Activated phosphor-PERK (upper) and total PERK (lower) indicated by arrow heads. (C) Quantification of PERK, p-eIF2a, JAG1 and DLL4 expression normalized to β-actin (right). (D-E) Representative EC (CD45^-^TER119^-^CD31^+^) expression of p-eIF2α (D) and ATF4 (E) by FACS analysis in control mice or leukemia mice with ICN1 T-ALL of 3 similar experiments. Data shown in MFI (D-E) were mean ± SD (n=4). Student t test was performed; *p<0.05; ** p<0.01.

We verified these findings by co-culturing ICN1 leukemia cells with both primary endothelial cells derived from bone marrow (BMECs) and immortalized mouse pancreatic ECs (MS1). Both JAG1 mRNA and protein expression were increased in BMECs exposed to leukemia (Fig. 1E & F) as well as in MS1 cells (Fig. S1). Cytokines important for HSC maintenance, such as CXCL12 and SCF, were down-regulated while VEGFα was up-regulated in co-cultured ECs. DLL4, another Notch ligand highly expressed by ECs 19, showed moderate increase in expression (Fig. 1E and below in Fig. 2). These findings indicate that T-ALL not only suppressed osteoprogenitor differentiation and HSPC proliferation 11 but also remodeled the vascular niche by altering the expression of hematopoietic cytokines/angiocrines and promoting angiogenesis. In addition, JAG1 up-regulation is a prominent feature of the T-ALL marrow vascular niche endothelium.

### PERK-eIF2a-ATF4 Activation induced in ECs by ICN1 is Necessary for JAG1 Up-regulation

Because recent findings indicated that acute myeloid leukemia (AML) activates mesenchymal stroma ER stress responses 20, we examined the role of UPR effectors in T-ALL ECs. We observed a significant increase of the shifted phosphorylated form of PERK in BMECs (Fig. 2A, top band; Fig. 2C, quantification) and increased PERK activation indicated by increased p-eIF2a in MS1 cells (Fig. S2) co-cultured with ICN1 cells compared to cells co-cultured with wild type (WT) BM cells. Because there was only mild increase in the expression of mRNA of total XBP1 (XBP1t) and no change of expression in spliced form of XBP1 (XBP1s) and activated transcription factor 6 (ATF6) (Fig. S3), we focused on the role of the PERK effector.

Increased phosphor-eIF2α (p-eIF2α) in BMECs accompanied increased PERK activation (Fig. 2A & C). Additionally, we observed an increase AT4 and CHOP transcription (Supplemental Fig. 3B). In addition to a marked increase of JAG1 (Fig. 2B and Supplemental Fig. 2), a moderate increase of DLL4 was observed (Fig. 2B-C). To investigate the significance of PERK induction by T-ALL *in vivo*, we examined the expression of p-eIF2 α, and ATF4 in ECs by flow cytometry. Consistent with *in vitro* findings, we found increased endothelial expression p-eIF2α (Fig. 2D, upper panels) and ATF4 (Fig. 2E, upper panels) in ICN1 mice compared to WT mice.

To assess if PERK pathway activation is responsible for JAG1 up-regulation, we treated ECs co-cultured with ICN1 cells with a PERK inhibitor (GSK2606414). PERK inhibition decreased p-eIF2a and JAG1 up-regulation in BMECs co-cultured with leukemia cells (Fig. 2A-C). To further investigate the role of PERK *in vivo*, we generated mice that are conditionally PERK deficient in ECs (VE-cadherin^ERT2-Cre^/PERK^F/F^) and assessed T-ALL development in these mice as well as the control PERK^F/F^ mice (Fig. S4A). PERK expression was significantly decreased, although not completely eliminated, in the bone marrow endothelium of the VE-cadherin^ERT2-Cre^/PERK^F/F^ mice (Fig. S4B). We found that BM endothelial increased p-eIF2α (Fig. 2D, lower panels) and ATF4 (Fig. 2E, lower panels) induced by leukemia was attenuated in the ECs of PERK-depleted mice. In comparison, PERK depletion in ECs had no significant effects on blood cell homeostasis in the periphery, spleen, and thymus (Fig. S5A-C). Additionally, numbers of homeostatic BM hematopoietic stem and progenitor cells (HSPCs), hematopoietic stem cells (HSCs), and total ECs were not affected by PERK depletion (Fig. S5D-F). These findings indicate that endothelial PERK activation mediates the activation of eIF2a-ATF4 and the up-regulation of JAG1 in T-ALL mice.

### ATF4 Regulates JAG1 While JAG1 Regulates Angiocrine Factor Expression

**Figure 3:**
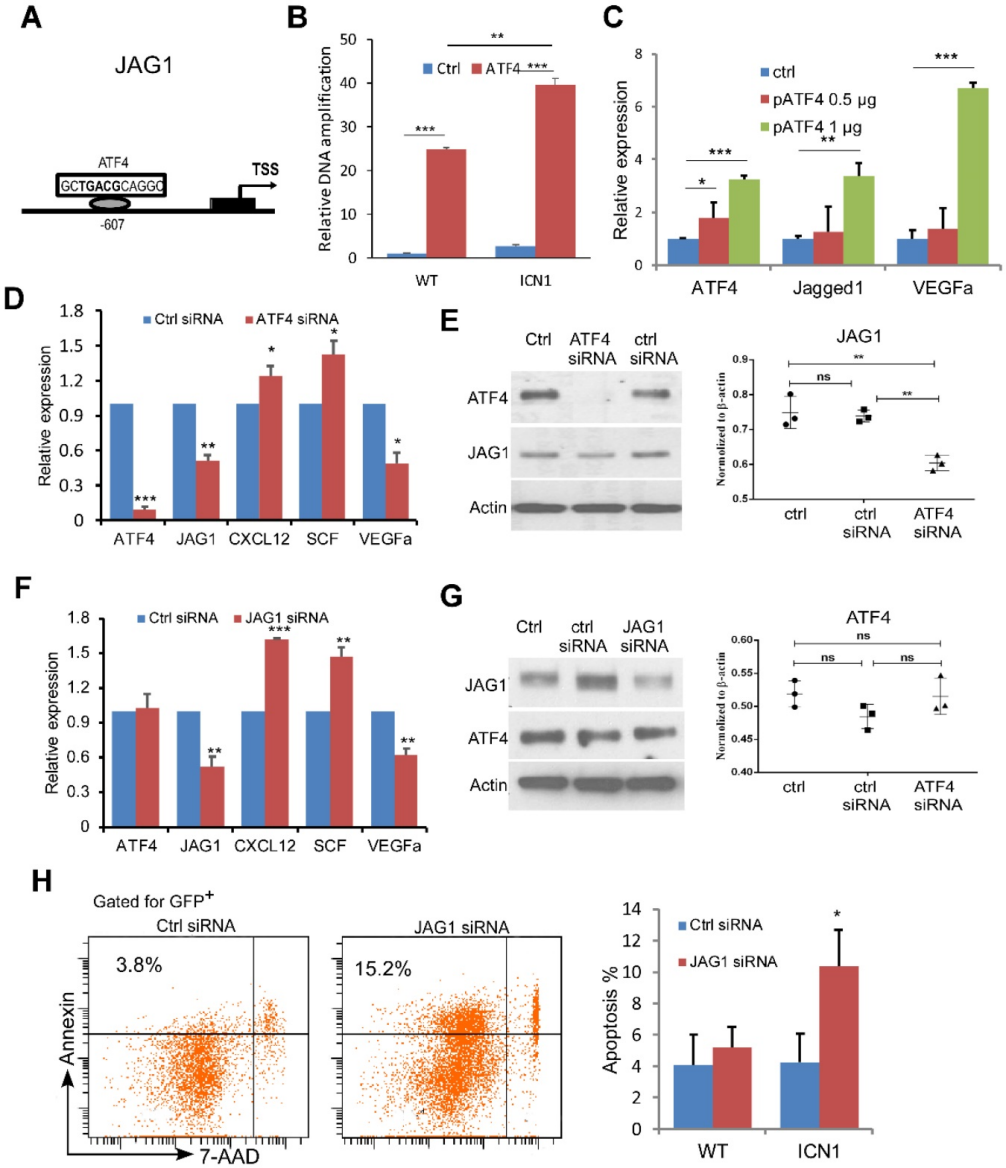
** ATF4 Regulates JAG1 While JAG1 Regulates Angiocrine Factor Expression.** (A-B) ChIP analysis with control rabbit IgG (ctrl IgG) and anti-ATF4 show JAG1 promoter region contains one CRE site approximately 0.6 kb upstream that was bound by ATF4 more in EC cells (MS1) exposed to ICN1 than in ECs exposed to WT bone marrow cells. (C-E) ATF4 over-expressing (C), ATF4 knockdown (D-E), or JAG1 knockdown (F-G) ECs were co-cultured with ICN1 cells (1.5 X10^6^) for 24h. qRT-PCR of transcripts were standardized for beta-actin and expressed as fold changes relative to those in ECs transfected with control siRNA or pcDNA plasmid (n=6/group from 2 experiments) (D & F). siRNA knockdown efficiencies and down-regulation of JAG1 after ATF4 knockdown were shown by western blots (E & G). (H) Representative FACS profile of apoptotic ICN1 cells co-cultured with ECs expressing control or JAG1 siRNA from 3 similar experiments. Data shown in right are mean percentage of apoptotic WT or ICN1 cells co-cultured with ECs expressing control or JAG1 siRNA (n=4). Data shown in B-H are mean ± SD. Student t test was performed; *p<0.05; ** p<0.01.

We next investigated the mechanism of JAG1 induction by PERK-eIF2a-ATF4. ATF4 binds to the cAMP response element (CRE) as a transcription activator and controls hundreds of potential target genes, including JAG1 21. To assess if ATF4 regulates JAG1 via the CRE binding site on the promoter region of JAG1 in the ECs (Fig. 3A) and if this regulation is influenced by T-ALL development, we performed chromatin immunoprecipitation (ChIP) in ECs co-cultured with WT or ICN1 cells. We found that ATF4 binds strongly to the CRE motif in the promoter region of JAG1. In addition, ECs co-cultured with ICN1 cells had higher level of bound ATF4 than those co-cultured with WT BM cells (Fig. 3B). Over-expression of ATF4 led to further increased JAG1 level (Fig. 3C) while down-regulation of ATF4 by siRNA decreased JAG1 level in ECs co-cultured with ICN1 leukemia cells (Fig. 3D-3E), indicating that EC JAG1 expression is regulated by ATF4 in the leukemia microenvironment. In addition, VEGFα down-regulation and up-regulation of CXCL12 and SCF were induced by the ATF4 knockdown (Fig. 3C) while increased expression of VEGFα accompanied with over-expression of ATF4 (Fig. 3D). A similar pattern of angiogenetic cytokine expression was observed by siRNA-mediated down-regulation of JAG1 (Fig. 3F) while JAG1 suppression had no effect on ATF4 expression (Fig. 3G). However, JAG1 down-regulation in ECs led to decreased leukemia cell survival (Fig. 3H). These findings indicate that ATF4 directly regulates JAG1, which in turn regulates HSCP-supporting cytokine expression and promotes leukemia survival.

### Targeting Endothelial PERK Increased HSPC Frequency and Induced Leukemia Cell Apoptosis

**Figure 4:**
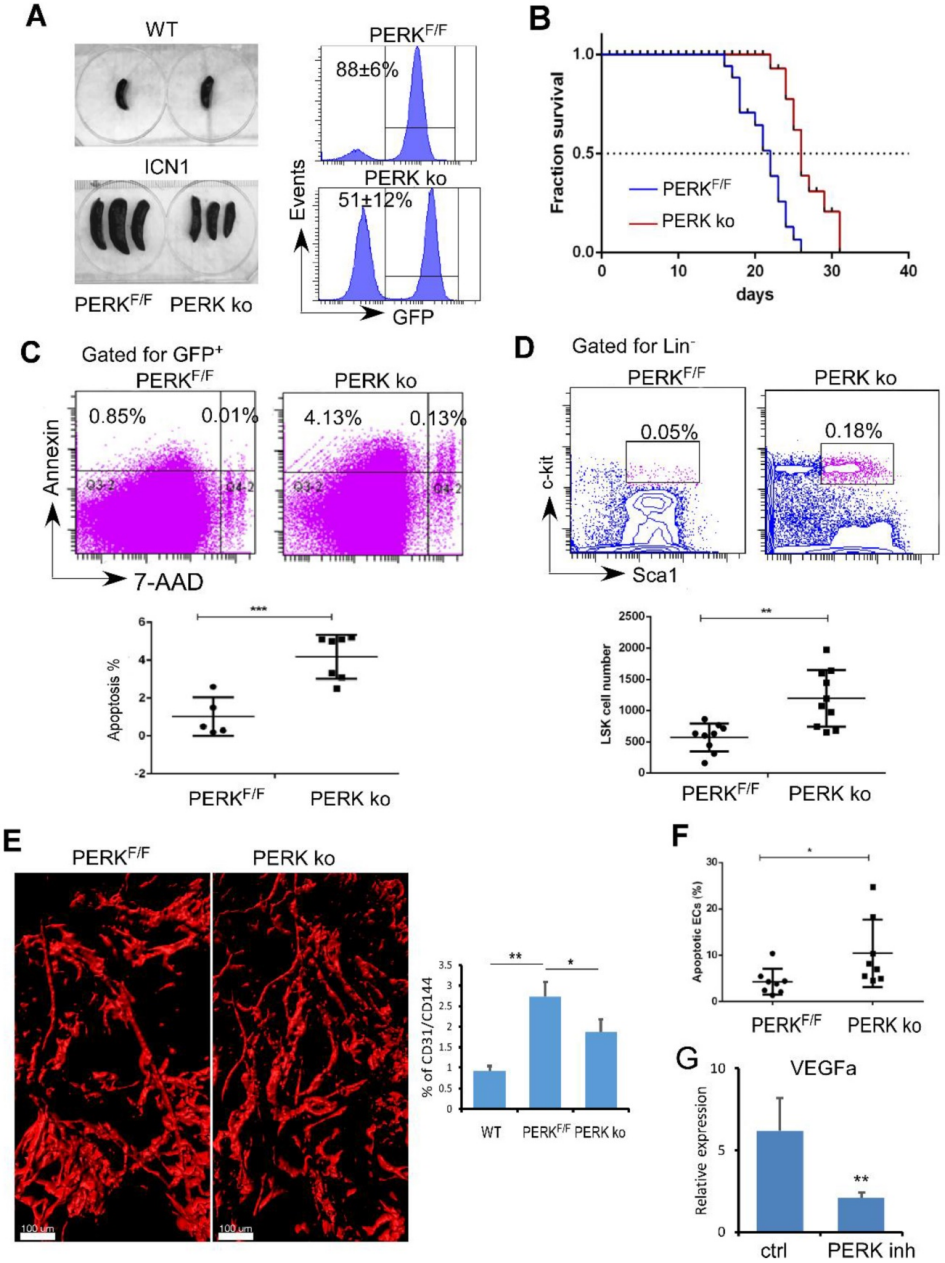
**Targeting Endothelial PERK Increased HSPC Frequency and Induced Leukemia Cell Apoptosis.** (A) Comparison of spleen sizes between WT mice (left top), ICN1control mice (PERK^F/F^), and ICN1-developing VE-cadherin^ERT2-Cre^/PERK^F/F^ mice (PERK-delpeted; PERK ko) mice (left bottom). Representative FACS profile of bone marrow GFP^+^ cells showing leukemia burden in PERK^F/F^ and PERK ko mice (right) from 3 experiments. (B) Kaplan-Myer survival curves of control leukemia mice (PERK^F/F^) (n=22) and ICN1-developing PERK knockout (PERK ko) (n=26) (pooled from 3 experiments). Mantel-Cox test was performed. p<0.0001. (C) Representative FACS profile of apoptotic annexin+ leukemia cells by gating on GFP+ cells in the marrow from 3 similar experiments. Means of apoptotic cells in control leukemia mice (PERK^F/F^) (n=9) and ICN1-developing PERK knockout mice (PERK ko) (n=10) were shown on the right. (D) Representative FACS profile of GFP^-^LSK and plots of frequencies in control leukemia mice (PERK^F/F^) (n=9) and ICN1-developing PERK knockout mice (PERK ko) (n=10) on day 14 after receiving primary leukemia cells. Data were pooled from 3 similar experiments. (E) Representative whole-mount imaging of CD31+ vasculatures and plots of CD31/CD144 volume (%) in PERK^F/F^ and PERK ko mice from 3 similar experiments. (F) Plots of apoptotic annexin^+^ ECs (CD45^-^TER119^-^CD31^+^) in control leukemia mice (PERK^F/F^) (n=8) and ICN1-developing PERK ko mice (n=8) on day 14 after receiving primary leukemia cells (top). (G) qRT-PCR of VEGFα expression in ECs co-cultured with ICN1 cells in the absence (ctrl) and the presence of PERK inhibitor (PERK inh) (n=6/group from 2 experiments). Data shown in C-F were mean ± SD. Student t test was performed; *p<0.05; ** p<0.01.

Previously we observed that T-ALL development depleted normal HSPCs 11. Here, we identified that PERK is activated in ECs upon leukemia development and down-regulates the production of angiogenic cytokines important for HSPC maintenance. We hypothesized that endothelial PERK activation remodels the vascular niche, suppresses residual hematopoiesis, and promotes leukemia progression. To test this, we assessed leukemia development and maintenance of residual HSPCs when PERK was depleted in VE-cadherin^ERT2-Cre^/PERK^F/F^ mice (PERK knockout; ko). Control recipients (PERK^F/F^) receiving leukemia cells from primary ICN1 mice developed secondary leukemia within 2 weeks while PERK-depleted mice showed reduced leukemia burden in the marrow and in the spleen (Fig. 4A). PERK-depleted mice displayed prolonged survival, with median survival extended from 22 days to 26 days (p<0.0001) (Fig. 4B). Improved survival in PERK-depleted mice was accompanied by increased leukemia cell apoptosis (Fig. 4C). Importantly, PERK-depleted mice had increased residual HSPCs in the marrow compared to control mice (Fig. 4D). The altered vascular architecture induced by ICN1 and the increased vessel density induced by leukemia were significantly reduced in PERK-depleted mice (Fig. 4E). The reversal of the enhanced angiogenesis was consistent with increased BM endothelial apoptosis upon PERK down-regulation (Fig. 4F) and decreased VEGFα expression in leukemia co-cultured ECs in the presence of PERK inhibitor (Fig. 4G). Combined with findings that PERK down-regulation suppressed PERK-eIF2a- ATF4 activation and JAG1 expression (Fig. 2), these observations suggest that endothelial down-regulation of PERK attenuated leukemia progression and improved survival by suppressing PERK-eIF2a- ATF4-JAG1 activation, thereby decreasing angiogenic factor expression, reducing leukemia cell survival, and preserving the residual hematopoiesis.

### Leukemia Extracellular Vesicles Induced PERK-dependent JAG1 Up-regulation

**Figure 5:**
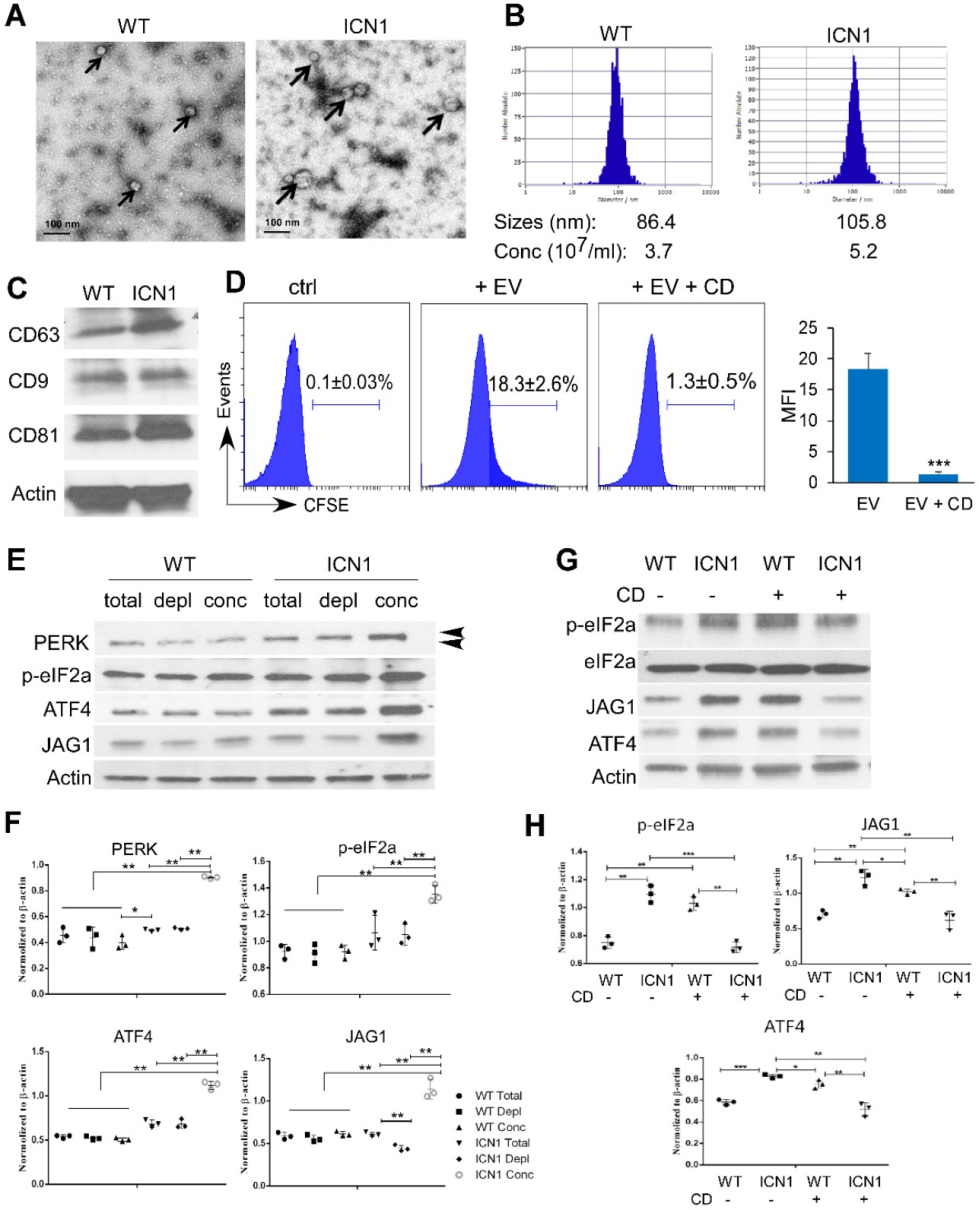
**Leukemia SEVs Induced PERK-dependent JAG1 Up-regulation.** Analysis of plasma-derived SEVs from control (WT) and ICN1 leukemia mice by electron microscopy imaging (A), nanoparticle analysis (B), and western blots of exosome markers (C). (D) CFSE-labeled SEVs were cultured with BMEC cells. Representative FACS profiles from 3 similar experiments showing CFSE signal when co-cultured with the SEV-depleted plasma (ctrl), CFSE-labeled ICN1 SEV (EV) (40 X10^8^/ml), or CFSE-labeled ICN1 SEVs together with CD (EV + CD). (E) Representative blots from 3 similar experiments of BMECs lysates after cultured for 24 h with whole plasma (total), concentrated exosomes isolated from plasma (conc) (40 X10^8^/ml), and exosome-depleted plasma (depl) collected from WT mice ICN1 mice peripheral blood. Activated phosphor-PERK (upper) and total PERK (lower) are indicated by the arrow heads. (F) Quantification of PERK, p-eIF2a, ATF4, and JAG1 expression normalized to β-actin. (G) Representative blots of BMECs lysates after cultured for 24h with concentrated exosomes isolated from WT mice or ICN1 mice in the absence or the presence of CD (5 µg/ml). (H) Quantification of p-eIF2a, ATF4, and JAG1 expression normalized to β-actin. Data shown in D was mean ± SD (n=3). Student t test was performed; *p<0.05; ** p<0.01.

To understand the mechanism by which T-ALL cells promote endothelial UPR response and JAG1 alteration, we examined the role of leukemia derived SEVs. SEVs are recognized as key mediators of cell-to-cell communication in many cancer types, including AML 22. To determine if T-ALL cells promote endothelial PERK activation through T-ALL-mediated SEVs, we prepared SEVs from the supernatant fractions of WT or ICN1 mice bone marrow and peripheral blood by high-speed centrifugation. Blood SEVs were used for the following studies because the quantity of bone marrow SEVs was insufficient for functional analysis. The presence of SEVs was confirmed by electron microscopy, nanoparticle tracking analysis, and western blot for exosome markers (CD63, CD9, and CD81) (Fig. 5A-C) 14, 15. The median size of circulating SEVs was 22% larger and the concentration of SEVs was 41% higher in leukemic mice compared to WT mice.

To determine if leukemia SEVs were taken up by ECs, CFSE-labeled SEVs were cultured with ECs. SEV internalization was observed in co-cultured BMECs (Fig. 5D). However, internalization was blocked by cytochalasin D (CD), a mycotoxin that disrupts actin cytoskeletal filaments and inhibits exosome up-take (Fig. 5D) 23, 24. We co-cultured BMECs with whole plasma collected from control mice (WT) or ICN1-developing mice, concentrated WT or ICN1 plasma SEVs, or SEV-depleted WT or ICN1 plasma. BMECs that were co-cultured with ICN1 plasma showed similar patterns of PERK-ATF4-JAG1 activation when compared to cells co-cultured with leukemia cells. Concentrated SEVs from ICN1 plasma induced a more pronounced up-regulation of JAG1 and activation of PERK-eIF2a-ATF4 (Fig. 5E). In comparison, SEV-depleted ICN1 plasma showed reduced activation of JAG1 but did not show significant change in the activation of the PERK-eIF2a-ATF4 axis when compared to the whole plasma, suggesting that, in addition to SEVs, other factors present in leukemic plasma contribute to PERK-eIF2a-ATF4 activation. To further test if the upregulation of JAG1 and the activation of PERK-eIF2a-ATF4 were mediated by SEVs, we treated co-cultured cells with CD (Fig. 5G-H). CD treatment in ECs exposed to control SEVs appeared to induce activation of eIF2α and ATF4 because of UPR-independent actin perturbation 25. In contrast, CD treatment inhibited eIF2a activation and suppressed ATF4 and JAG1 up-regulation induced by leukemia SEVs (Fig. 5F). The cause of these discrepant results by CD treatment remains unclear. To confirm the role of SEVs in the induction of JAG1, we took an alternative approach and treated ICN1-exposed ECs with GW4869. GW4869 blocks inward budding of multivesicular bodies and the release of mature exosomes 26, 27. Similar to CD treatment, GW4869 partially decreased the up-regulation of JAG1 and the activation of eIF2α mediated by control cells, but it blocked the activation of eIF2a, ATF4, and JAG1 mediated by ICN1 cells (Fig. S6). These findings suggest that T-ALL associated SEVs induce endothelial PERK-ATF4-JAG1 activation.

### Endothelial PERK Activation and JAG1 Over-expression in Human T-ALL

**Figure 6:**
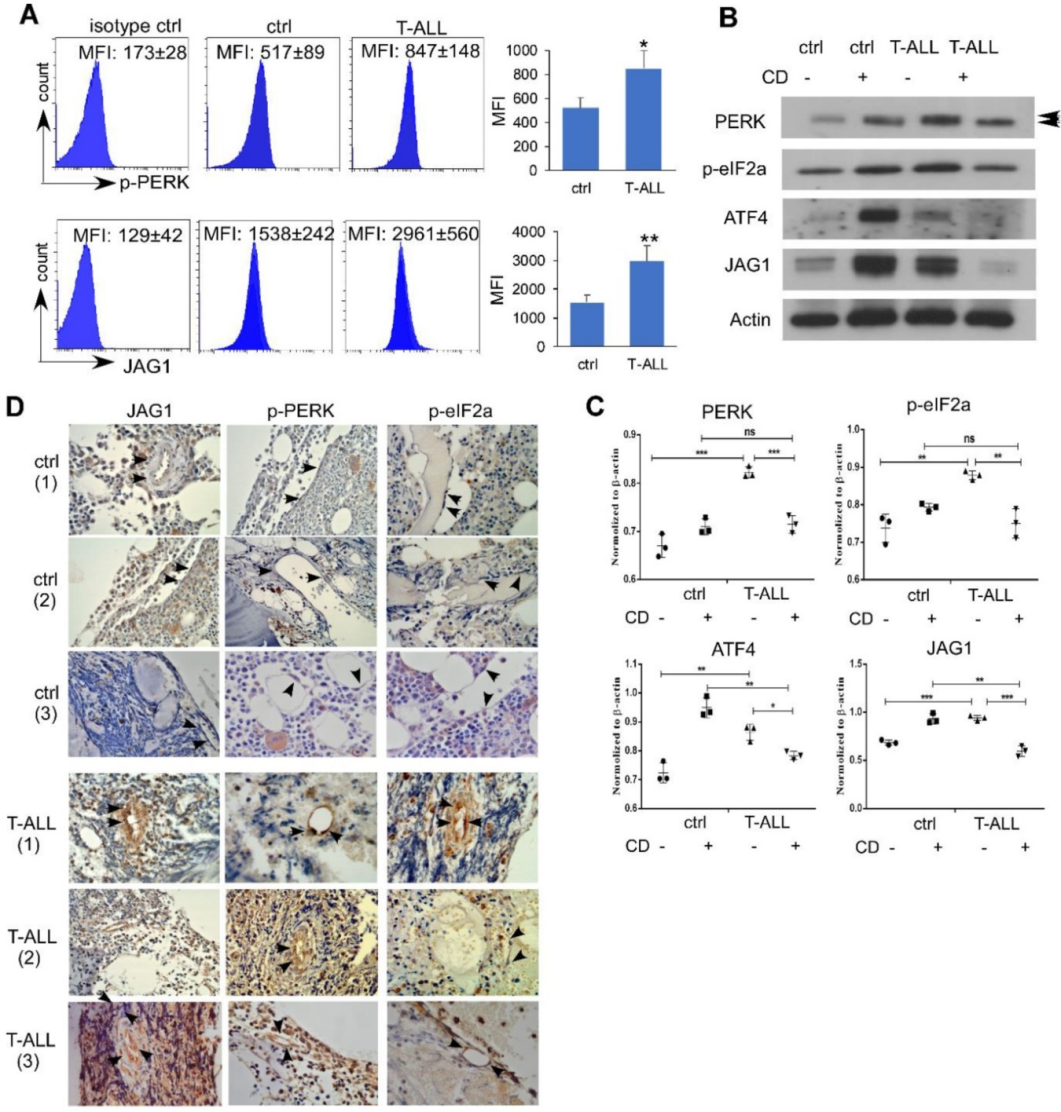
**Endothelial PERK Activation and JAG1 Over-expression in Human T-ALL.** (A) NSG mice were IV injected with PBS (ctrl) or engrafted with human DND41 cells in 3 independent experiments. Marrow leukemia burden was determined by the expression of human CD45 (hCD45). Representative FACS profiles of host p-PERK and JAG1 in control mouse or DND41-engrafted mouse ECs (hCD45^-^). (B) Representative western blots of MS1 cells after being cultured with DND41 leukemia cells in the presence or the absence of CD from 3 similar experiments. (C) Quantification of PERK, p-eIF2a, ATF4, and JAG1 expression normalized to β-actin. (D) IHC staining of PERK, p-eIF2α, and JAG1(arrows) in human bone marrow tissues from two non-neoplastic (ctrl) or T-ALL cases. Images were taken under 200X magnification. Data shown in A are mean ± SD (n=5 from 3 experiments). Student t test was performed; *p<0.05; ** p<0.01.

To examine if human T-ALL exhibits similar EC alterations, we engrafted NSG mice with human T-ALL DND41 cells. Like mice developing ICN1, NSG mice engrafted with DND41 showed increased endothelial expression of p-PERK and JAG1 (Fig. 6A). *In vitro* co-culture of DND41 and ECs induced PERK, eIF2a and ATF4 activation accompanied by JAG1 up-regulation. In addition, the activation of the UPR effectors and the up-regulation of JAG1 by T-ALL was suppressed by CD while CD induced an UPR-independent activation of these effectors in cells co-cultured with WT cells (Fig. 6B-C).

To determine the relevance of our findings in T-ALL patient bone marrow vascular niche, we examined sampled non-leukemic human BM sections and sections from T-ALL patients. ECs were identified by morphology. Compared to non-leukemia tissues (Fig. 6D), JAG1 expression was increased in all T-ALL marrow sections that exhibited stronger cytoplasmic /membrane staining of ECs. Activated PERK and ATF4 showed strong nuclear expression in T-ALL specimens but not in control cases. These findings are consistent with animal models and human T-ALL grafts and support a prominent activation of endothelial PERK- eIF2a-ATF4-JAG1 in T-ALL.

## Discussion

UPR has emerged as a key player for highly proliferating cells, including leukemia cells 28, 29. Using a mouse T-ALL model, we found that activated Notch1 is associated with up-regulation of JAG1 in marrow ECs under the direct regulation ATF4 through the PERK effector of UPR. Leukemia-induced JAG1 up-regulation in ECs led to altered expression of HSC-supporting cytokines, including SCF, CXCL12, and the angiocrine factor VEGFα. T-ALL development increased bone marrow vessel density, which could be reversed by endothelial down-regulation of PERK. In addition, targeting endothelial PERK increased residual HSPCs and improved animal survival via leukemia cell apoptosis. Importantly, we found that T-ALL exosomes induced PERK-ATF4-JAG1 activation in ECs. Depleting exosomes or blocking exosome uptake/release abolished JAG1 induction. To support animal model studies, we found BM endothelial activation of PERK-ATF4-JAG1 is a prominent feature in human T-ALL tissues and xenografts. Thus, our findings reveal a novel mechanism underlying leukemia remodeling of HSC vascular niche through induction of endothelial UPR.

UPR is critical for meeting the increased demand for protein synthesis required for rapid cellular proliferation and for adapting to pathological perturbations in the leukemia microenvironment, such as pH changes, nutrient deprivation, and increased levels of reactive oxygen species. Among the three conserved stress sensors (inositol-requiring enzyme-1α (IRE1α), PERK, and ATF6), PERK was most significantly activated in the presence of T-ALL cells. Severe and continuous ER stress activates PERK to phosphorylate eIF2 to repress global translation except for a few genes, such as ATF4, whose mRNAs contain short open reading frames in their 5'-untranslated regions. ATF4 regulates genes involved in apoptosis, such as CHOP, or restoring ER homeostasis. In both AML and ALL, UPR activation leads to elimination of misfolded proteins and apoptosis resistance through various mechanisms, such as enhancement of the ER-associated degradation (ERAD) and IRE1α-dependent decay (RIDD) responses 30-33. Following our previous reports that aberrant Notch activation inhibits the expression of CXCL12 on osteoblasts and suppresses osteoprogenitor differentiation, we found that T-ALL induced JAG1 up-regulation in ECs through a PERK-ATF4-dependent process.

ECs are important components of the bone marrow niche, which includes osteoblasts, adipocytes, and perivascular stromal cells 34-37. ECs secrete SCF, CXCL12, and other growth factors important for HSC maintenance and regeneration 35, 38. Targeting endothelial PERK not only improved HSC generation but also promoted leukemia cell apoptosis. The protective effects on hematopoiesis were accompanied by increased expression of CXCL12 and SCF, whose expression was suppressed by infiltrating leukemia cells. Although CXCL12 expressed by ECs is required for T-ALL expansion in mice 39, its leukemia-promoting effect is probably antagonized by other cytokines and angiocrine factors that can be restored by PERK inhibition. For example, leukemia development induced up-regulation of the angiocrine factor VEGFα in addition to JAG1. VEGFα confers survival benefits on leukemia cells in chronic lymphocytic leukemia (CLL) through autocrine and paracrine mechanisms 40, 41. Elevated level of VEGFα is correlated with BM angiogenesis and increased BM micro-vessel density in ALL patients 42, 43. This is consistent with our findings that ICN1 mice display increased CD31^+^ vessel density both by flow and by whole-mount imaging. We showed that increased endothelial density and increased VEGFα were suppressed by PERK deletion, which also led to increased endothelial cell apoptosis. Thus, in addition to the internal cellular response that promotes cellular survival 30-33, we have identified a novel endothelial-specific PERK-regulated mechanism that couples enhanced angiogenesis, leukemia survival, and compromised HSPC regeneration in the leukemia vascular niche.

We previously observed significant osteoblast loss and associated Notch activation in human T-ALL xenografts and human ALL specimens. Specifically, osteoprogenitor differentiation was negatively regulated by Notch activation and stroma JAG1 up-regulation.^11^ Here, we further explored the mechanism underlying the aberrant JAG1 expression in the leukemia-associated vascular niche in mouse models and in the human T-ALL BM microenvironment. We revealed that JAG1 is a downstream molecule of PERK activation and is directly regulated by ATF4. Down-regulation of ATF4 partially inhibited JAG1 up-regulation and down-regulation of JAG1 recovered suppressed SCF and CXCL12 expression, attenuated VEGFα induction, and induced leukemia cell apoptosis. These observations suggest that JAG1 is an important node for HSC-supporting cytokines and angiogenesis-promoting factors in the leukemia vascular niche, although the molecular pathways of aberrant cytokine expression and angiocrine factor expression controlled by JAG1 and other transcription factors that may regulate JAG1 activation remain to be determined. In tumor models, JAG1 plays an important role in promoting sprouting angiogenesis and antagonizing DLL4-mediated Notch signaling 44-47. In addition, JAG1 promotes cancer stem cell self-renewal, tumor cell proliferation, drug resistance and survival 48. In solid tumors, JAG1 play a role in the promotion of cancer stem cell phenotype by promoting both pro-angiogenic and angiocrine functions in ECs 49, 50 Our findings provide strong rationale for future studies that explore the potential of targeting endothelial JAG1 to suppress leukemia cell survival and promote residual hematopoiesis in T-ALL patients.

SEVs have been extensively studied in AML progression and the rewiring of the leukemia microenvironment 22. However, SEV and SEV trafficking have not been reported in T-ALL BM microenvironment as a significant signaling paradigm. We confirmed a viable communication between ECs and co-cultured T-ALL cells through SEVs by showing that active uptake of labeled leukemia SEVs could be blocked by the EV biogenesis/release inhibitor. Importantly, we showed that endothelial PERK-ATF4-JAG1 activation and altered angiocrine factor expression in the presence of leukemia cells was significantly inhibited by depleting SEVs or blocking SEV transmission. Our findings suggest that leukemia SEV transmission could be a conduit linking leukemia progression and PERK activation in the leukemia vascular niche, although the SEV content responsible for these activities is currently unknown and warrants further study. The leukemia bone marrow is a complex microenvironment where oncogenic genetics and a surrounding malignant niche with cellular and non-cellular elements converge. Therefore, a comprehensive dissection of the molecular mechanism regulating the leukemia vascular niche is limited by the co-culture platform employed in this study.

It has been well documented that direct interaction between leukemia cells and the bone marrow micro-environment through adhesion molecules regulate leukemia cell homing, survival, and metastasis 51. Integrins, for example, are important cell adhesion receptors that engage in T-ALL anti-apoptotic signaling when binding extracellular matrix ligands enriched in the bone marrow stroma cells 52, 53. We examined the expression of VCAM-1 and ICAM-1 in the marrow vascular niche of normal and ICN1 leukemia mice. We found that VCAM-1 and ICAM-1 were expressed in normal bone marrow ECs, but mice engrafted with ICN1 leukemia exhibited increased expression of both adhesion molecules (Fig. S7). However, PERK depletion had no effect on ICAM-1 or VCAM-1 expression. To investigate if the adhesion between leukemia cells and ECs induces EC UPR response and PERK activation, we blocked the ligands of ICAM-1 and VCAM-1 and found that PERK, eIF2a, and JAG1 activation was modestly attenuated by blocking LFA-1 but largely unaffected by blocking VLA-4 (Fig. S8A-B). Consistent with other reports, we found that both LFA-1 and VLA-4 blockade promoted leukemia apoptosis; however, PERK inhibitor induced even more leukemia cell apoptosis (Fig. S8C). These findings suggest that increased adhesion of ICN1 leukemia cells to ECs through LFA-1/ICAM-1 but not VLA-4/VCAM-1 may contribute to the endothelial UPR induction and leukemia bone marrow remodeling while PERK depletion restored niche function independently of these adhesion processes. The specific role and the underlying mechanism of UPR induction by ICAM-1 and possibly other adhesion molecules would require further investigation, which may be performed through genetically engineered mouse models 54. In addition, it would be important to assess whether targeting adhesion molecules combined with targeting endothelial PERK or JAG1 can synergistically suppress T-ALL survival and impact leukemia progression. Although targeting PERK *in vitro* by GSK260414 was effective, *in vivo* application of this PERK inhibitor in ICN1 mice resulted in lethality likely due to a combination of off-target toxicity (e.g. RIPK1 and c-Kit) and organ toxicity (e.g., pancreatic toxicity) that is enhanced in the setting of leukemia 55-57. Developing PERK-specific and less toxic inhibitor is necessary for its successful application in cancer treatment in the future.

In summary, our results demonstrated a previously unidentified endothelial PERK-ATF4-JAG1 axis in the remodeling of the marrow vascular niche and supported the notion of niche restoration as additional therapeutic opportunities to improve on current T-ALL treatments.

## Supplementary Material

Supplementary figures.
