# Supplementary material for: Endothelial PERK-ATF4-JAG1 axis activated by T-ALL remodels bone marrow vascular niche
Source: Theranostics. 2022 Mar 21;12(6):2894–907. doi: 10.7150/thno.67710 (PMC8965499; doi:10.7150/thno.67710)
Supplement: Supplementary file 1 — Supplementary figures. [file thnov12p2894s1.pdf]

Supplemental figure legneds and figures

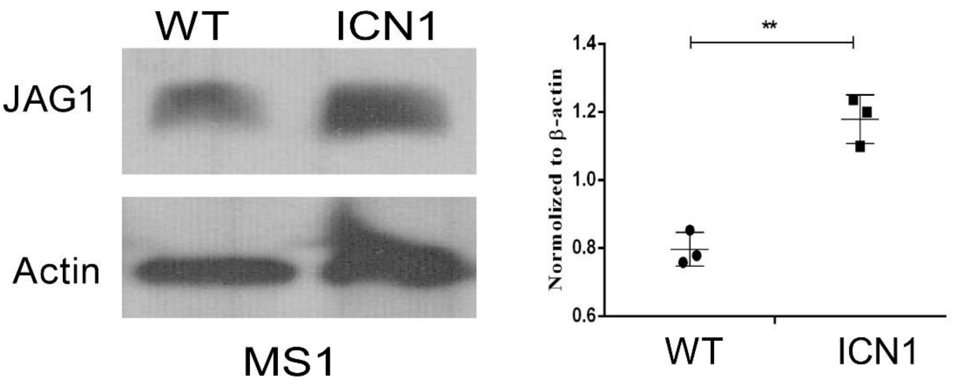

Fig S1. Up-regulation of JAG1 in MS1 cells. MS1 cells were co-cultured with control or ICN1 cells (1.5 X10^6) for 24h. Representative western blot (left) with anti-JAG1. Quantification of JAG1 expression normalized to β-actin from 3 similar experiments (right). Student t test was performed; **p<0.01

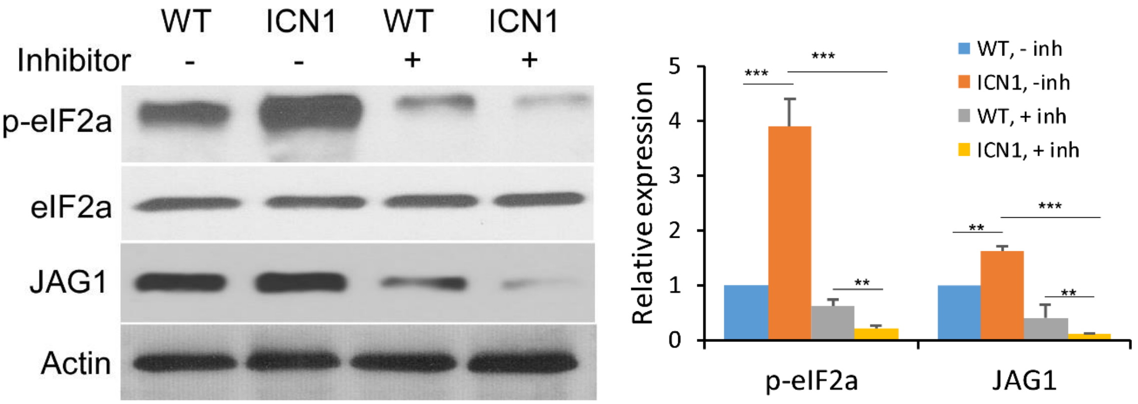

Figure S2. PERK and JAG1 Activation induced in MS1 by ICN1. MS1 cells were co-cultured with control or ICN1 cells (1.5 X10^6) for 24h in the presence or absence (GSK control medium) of the PERK inhibitor GSK2606414 (GSK; 1 μM). Representative western blots using antibodies targeting p-eIF2a, eIF2a, JAG1, and actin were shown of 3 similar experiments.

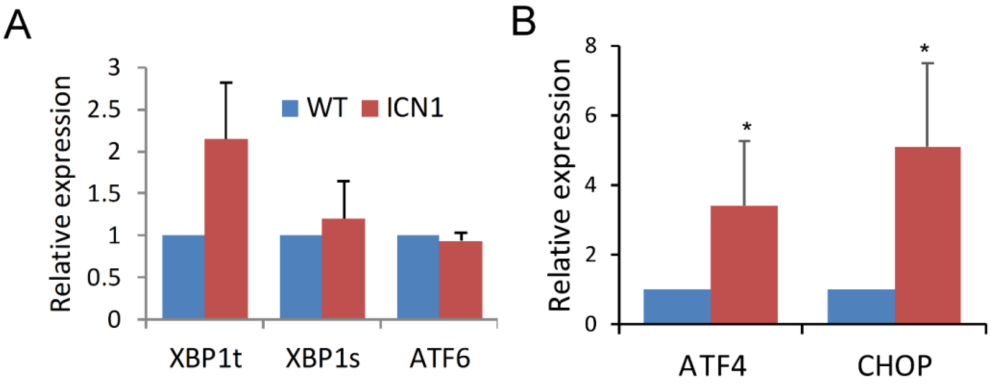

Fig S3. Endothelial UPR effector regulation by ICN1. BMECs were co-cultured with control or ICN1 cells (1.5 X10^6) for 24h. qRT-PCR of EC expressions of the UPR effectors (total XBP1 (XBP1t), spliced XBP1 (XBP1s) and ATF6) (A) and ATF4 & CHOP (B) were standardized for beta-actin and expressed as fold changes relative to those in EC co-cultured with control marrow cells (n=6/group from 2 experiments). Student t test was performed; *$p<0.05$

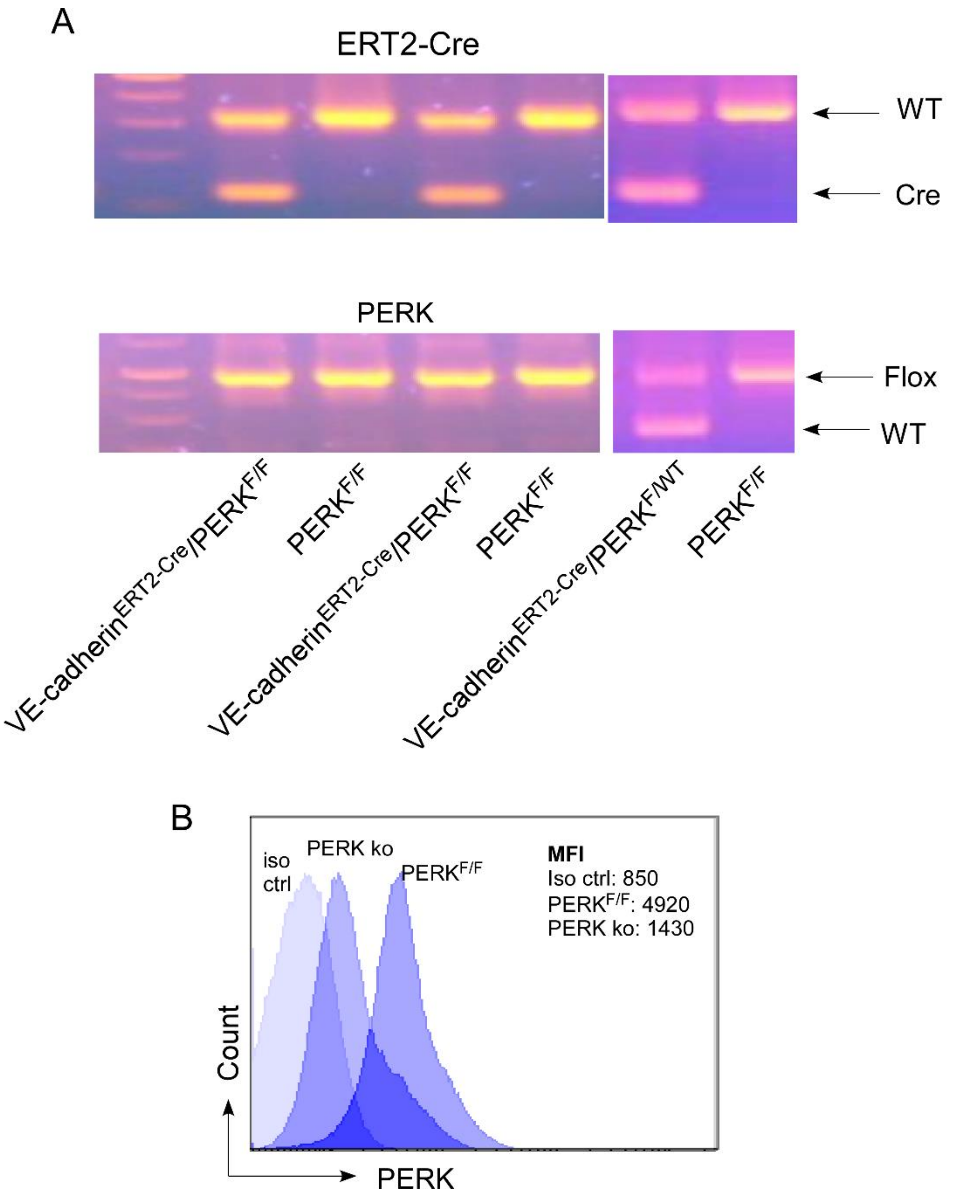

Fig S4. Genotyping of VE-cadherin$^{ERT2-Cre}$/PERK$^{F/F}$ mice and control mice. (A) Genotyping of ERT2-cre and PERK floxed allele in VE-cadherin$^{ERT2-Cre}$/PERK$^{F/F}$ and PERK$^{F/F}$ mice. (B) Detection of PERK expression (BS-2469R; Bioss Antibodies Inc., Woburn, MA) by flow bone marrow ECs (gated on CD45$^-$TER119$^-$CD31$^+$ cells) in PERK knockout (ko) (VE-cadherin$^{ERT2-Cre}$/PERK$^{F/}$F) and PERK$^{F/F}$ mice.

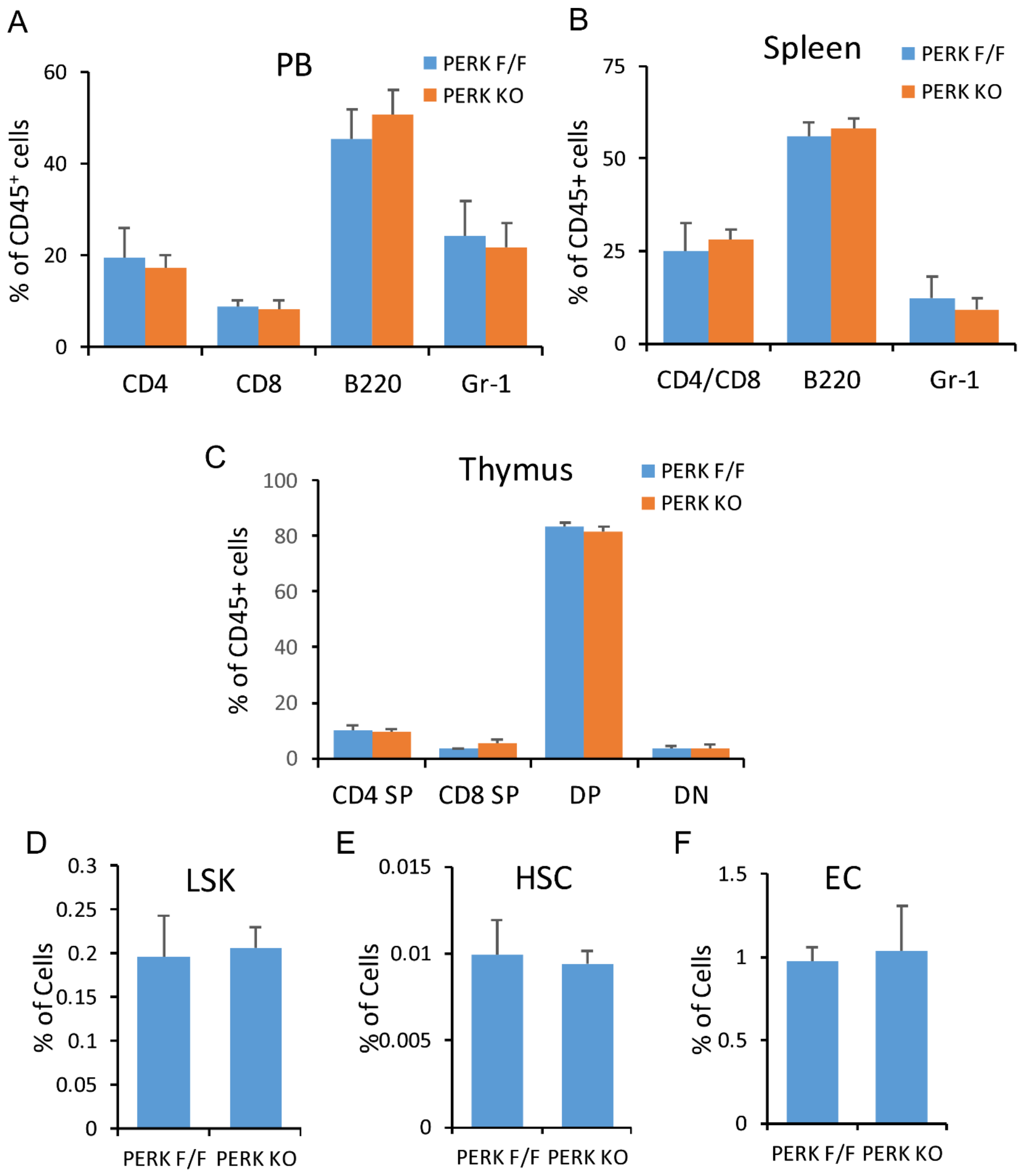

Fig S5. PERK deletion in endothelial cells had no significant effects on homeostatic hematopoiesis. One month after PERK deletion by tamoxifen, peripheral blood (PB) (A), spleen (B), thymus (C) and bone marrow (D) were analyzed for PB and spleen T cells (CD4+ or CD8+), B cells (B220+), granulocytes (Gr-1+); thymus CD4+ (CD4 SP), CD8+ (CD8+ SP), CD4+CD8+ (DP), and CD4-CD8- (DN); bone marrow HSPC (lin-Sca1+c-kit+; LSK), HSC (CD4-Flt3-CD150+CD48-LSK), and endothelial cells (CD45-TER119-CD31+; EC). Data pooled from 8 mice of each genotype and expressed as mean ± SD. Student t test was performed

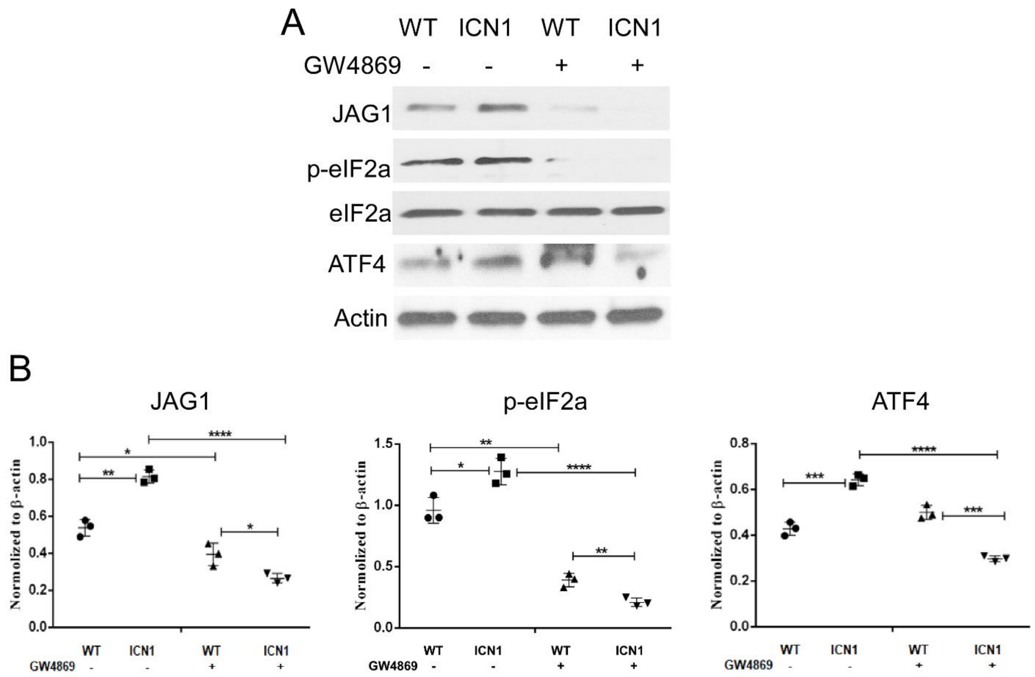

Fig S6. Inhibition of SEV biogenesis/release blocked leukemia induced JAG1 up-regulation. (A) Representative blots of BMECs lysates after cultured for 24 h with control or ICN1 cells in the absence or the presence of GW4869 (20 µmol/L). (B) Quantification of total eIF2a, p-eIF2a, ATF4, and JAG1 expression were normalized to β-actin. Data shown in B was mean ± SD (n=3). Student t test was performed; *p<0.05, ** p<0.01, *** p<0.001, **** p<0.0001

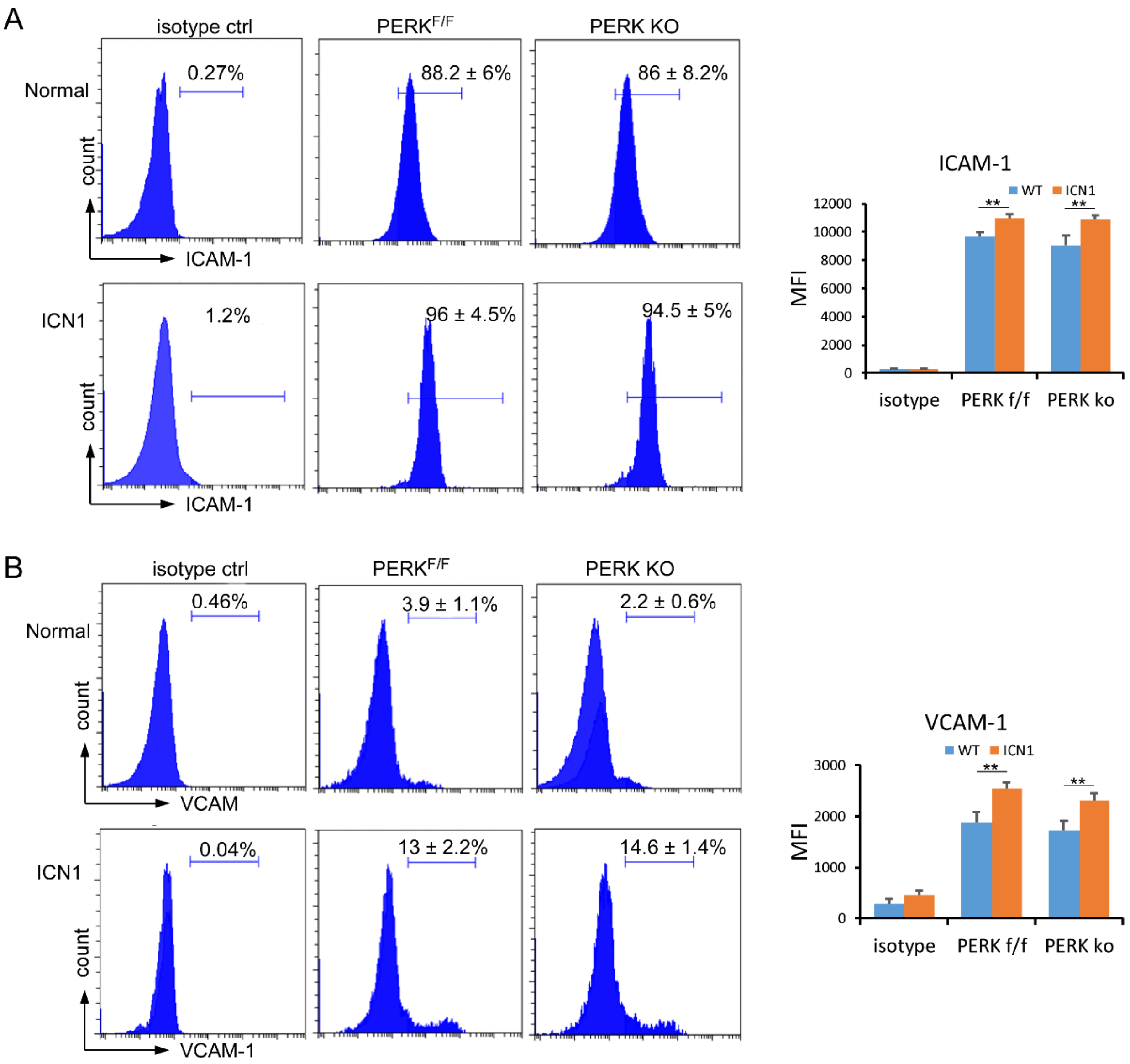

Fig S7. ICAM-1 and VCAM-1 expression levels in the bone marrow endothelial cells. (A-B) Representative FACS profile of bone marrow EC ($Lin^{-}TER119^{-}CD31^{+}$) expression of ICAM-1 (A) and VCAM (B) in normal mice and mice engrafted with ICN1 T-ALL cells. Leukemia burden was >85% in all leukemia developing mice. Data shown are mean ± SD (n=3/group).

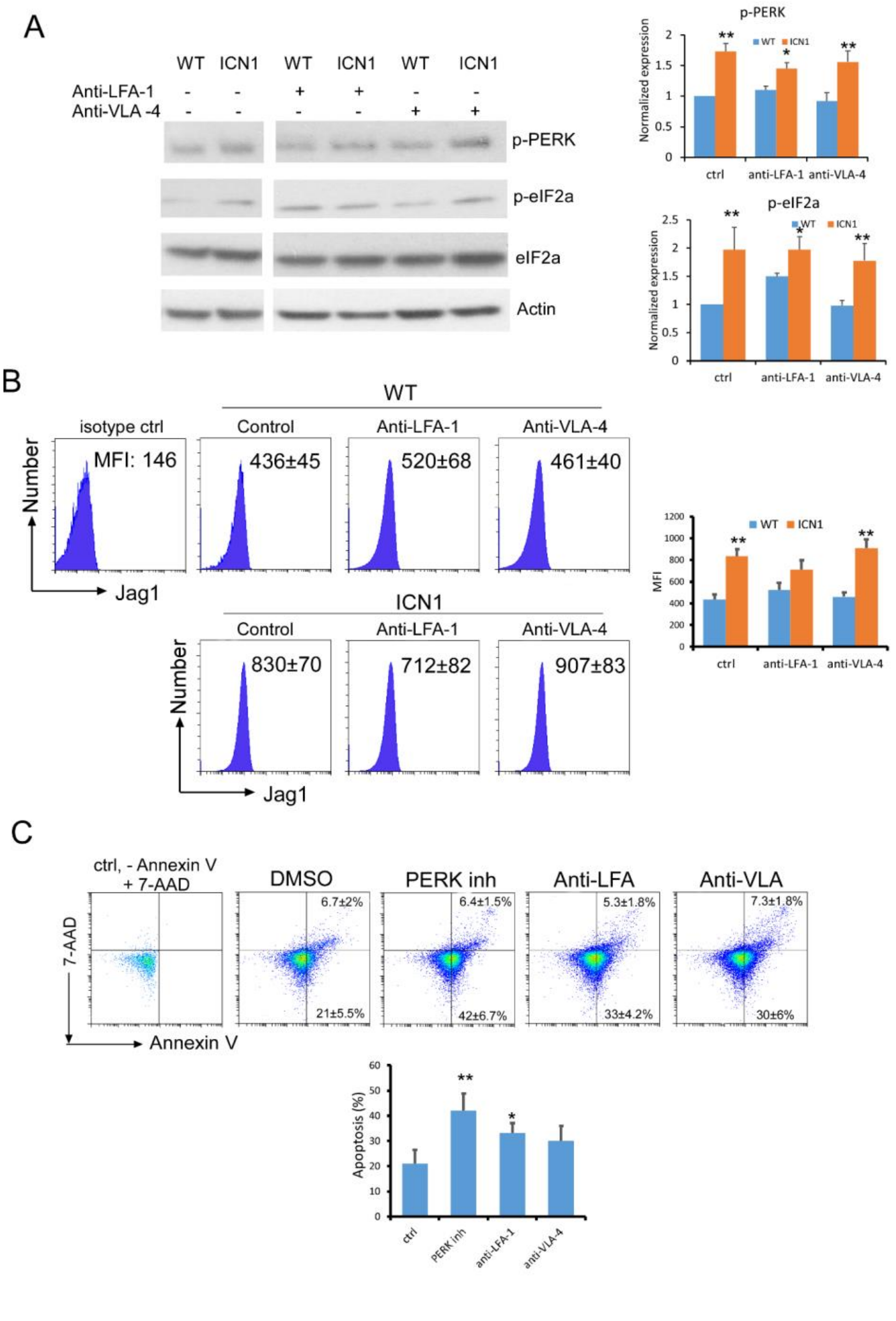

Fig S8. LFA-1/ICAM-1 may contribute to PERK and JAG1 activation in endothelial cells. (A-B) Representative blots of PERK and eIF2a activation in EC lysates (A) and flow profile of JAG1 expression (B) after cultured for 24 h with control or ICN1 cells in the absence or the presence of anti-LFA-1 (10 µg/mL) (BE0005-1; BioXCell) or anti-VLA-4 (10 µg/mL) (BE0071; BioXCell). Quantification of p-PERK (MA5-15033; Invitrogen) and p-eIF2a was normalized to β-actin. The expression of WT p-PERK or p-eIF2a to β-actin was set as 1. (C) Apoptotic ICN1 cells (annexin-V$^+$7-AAD$^-$) were assessed by annexin-V staining after co-culture with ECs for 24 h with DMSO (ctrl), PERK inhibitor, anti-LFA-1, or anti-VLA-4. Data shown are mean ± SD (n=3/group). Student t test was performed; *p<0.05, ** p<0.01.
